# Dynamics of Postmortem Gene Expression in Normal and Neoplastic Murine Liver

**DOI:** 10.3390/life16040683

**Published:** 2026-04-16

**Authors:** Evgeny E. Buyko, Ekaterina A. Perina, Danil S. Sobakin, Matvey M. Tsyganov, Dmitry V. Vasilchenko, Sergey V. Vtorushin, Alexander A. Ufandeev, Elena B. Diksas, Olga A. Kaidash, Ekaterina S. Hmelevskaya, Ekaterina V. Parochkina, Igor A. Popov, Vladimir V. Ivanov, Stanislav I. Pekov, Elena V. Udut

**Affiliations:** 1 Central Research Laboratory, Siberian State Medical University, 634050 Tomsk, Russia; buykoevgen@yandex.ru (E.E.B.); tsyganovmm@yandex.ru (M.M.T.); kaidash_2011@mail.ru (O.A.K.); ivanovvv1953@gmail.com (V.V.I.); 2 Laboratory for Molecular Medical Diagnostics, Moscow Institute of Physics and Technology (National Research University), 141701 Dolgoprudny, Russiahexapole@gmail.com (I.A.P.)

**Keywords:** gene expression, postmortem interval, hepatocellular carcinoma, experimental model, diethylnitrosamine, thioacetamide, RNA quality number

## Abstract

The use of postmortem (autopsy) material in fundamental and applied biomedical research significantly facilitates the collection of biomaterial for statistically robust sample cohorts. However, natural adaptive processes to developing cellular stress in the early postmortem period, caused by oxygen and nutrient deprivation, trigger the activation of numerous genes promoting cell survival under stress. Many of these activated pathways are also crucial for tumor cell survival in vivo, as evidenced by various transcriptomic studies. This study aimed to investigate the potential influence of postmortem interval (PMI) duration on gene expression in normal and tumor tissues. Using a model of chemically induced hepatocellular carcinoma in mouse liver, we comparatively analyzed the dynamics of transcript levels for several genes (BRCA1, BRCA2, CHEK1, CHEK2, ATM, CDK12) in paired samples of normal and tumor tissue over a 24-h PMI using RT-qPCR. In normal tissue, gene expression increased significantly, while tumor tissue demonstrated relative transcriptional stability, with no substantial changes in the studied transcript levels. A critical finding was the observed convergence of expression profiles: initial differences between the tissues were completely eliminated by 24 h PMI. This pattern developed despite formally adequate RNA quality (RQN) and the absence of clear signs of progressive autolysis in histology, indicating the insufficiency of standard quality criteria for detecting postmortem changes. These findings collectively underscore the critical importance of minimizing and controlling PMI during the biobanking of oncological samples for reliable transcriptomic research.

## 1. Introduction

The use of postmortem biological specimens constitutes a cornerstone of modern biomedical research, particularly in oncology, neurodegenerative, and cardiovascular diseases [1,2]. Biobanks incorporating autopsy-derived tissue samples provide invaluable material for studying disease pathogenesis, discovering novel biomarkers, and identifying therapeutic targets. A fundamental limitation compromising the reliability of such studies, however, is the profound yet unpredictable influence of the postmortem interval (PMI)—the time elapsed between death and tissue preservation—on tissue integrity and molecular profiles [3,4]. Autopsy material is also of great interest for improving the accuracy and specificity of novel molecular-profiling-based tools designed to assist surgeons in decision-making [5].

RNA stability is a central concern in molecular biological analysis, being critically important for accurate gene expression assessment. Numerous studies have demonstrated that PMI significantly and often tissue-specifically impacts RNA degradation and, consequently, transcript levels. For instance, in human brain tissue, increased PMI has been correlated with a decline in RNA quality number (RQN) and a reduction in the transcript levels of most studied genes [3]. Similarly, a large-scale analysis of data from the GTEx project revealed tissue- and gene-specific patterns of mRNA degradation with increasing PMI, highlighting the complexity and context-dependent nature of this phenomenon [4]. These findings present a major methodological quandary: how to distinguish genuine, physiologically or pathologically driven changes in gene expression from artifacts introduced by postmortem processes.

This challenge has given rise to the field of thanatotranscriptomics, which investigates global changes in gene expression following organismal death [6,7,8]. Contrary to the intuitive assumption of an immediate cessation of all biological processes, research has demonstrated that transcriptional activity can persist in tissues for a considerable amount of time [9]. Moreover, these postmortem changes are not random but often constitute a concerted cascade of events. In mouse and zebrafish tissues, the relative abundance of transcripts from over 1000 genes significantly increased for up to 96 h postmortem, with these genes being functionally linked to stress response, immunity, inflammation, and apoptosis [7]. Beyond transcriptomics, the stability of the metabolome postmortem is also a subject of active investigation, as evidenced by large-scale studies showing significant time-dependent and species-specific alterations in lipid and polar metabolite levels in the brains of humans and rodents [10].

Genes responsible for DNA repair and cell cycle control are of particular interest in the context of cancer and, furthermore, in postmortem changes [11,12,13]. DNA damage is a key stressor induced by postmortem hypoxia and ischemia. It is logical to hypothesize that cells would attempt to activate mechanisms to repair their genetic material. Scattered scientific reports support this notion. For instance, analysis of postmortem human blood cells revealed the activation of pathways promoting cell survival and DNA repair [6]. Another study provided evidence that genes associated with the response to oxidative stress and hypoxia are activated in postmortem human skeletal muscle [14]. Thus, the postmortem activation of repair genes in normal tissues appears to be a plausible adaptive response to critical damage.

Despite significant progress in studying postmortem gene expression, the overwhelming majority of research has focused on normal, pathologically unaltered tissues [1,4,10]. Meanwhile, a primary source of biomaterial for oncological research consists of samples obtained during autopsies or surgical procedures, inevitably with a delay between the cessation of blood supply and cryopreservation [2,15,16]. This discrepancy creates a substantial methodological gap.

Tumor cells are fundamentally distinct from their normal counterparts in terms of biology. They are characterized by chronic genomic stress due to oncogenic mutations and elevated oxidative damage; impaired apoptosis and cell cycle control; reprogramming of energy and lipid metabolism; and constitutive activation of numerous survival signaling pathways [2,17]. It can be hypothesized that these fundamental differences should also dictate a distinct response of tumor tissue to postmortem stress compared to normal tissue. However, to date, systematic studies dedicated to the direct comparative analysis of gene expression dynamics in paired samples of normal and tumor tissue over an extended postmortem period are virtually nonexistent. Only a few works indirectly touch upon this issue. For example, Gupta et al. (2012) demonstrated that the transcriptional profile of human heart tissue remains remarkably stable over 24 h of autolysis, with only a small fraction of genes (<2.5%) showing altered expression [1]. The authors concluded that RNA from autopsy tissue, even after 24-h of autolysis, could be used to detect biologically significant differences. This raises a pivotal question: would tumor tissue, with its inherently different regulatory landscape compared to normal tissue, demonstrate similar stability or, conversely, increased lability?

Another crucial consideration pertains to the selection of appropriate controls in oncological research. Tissue adjacent to the tumor is often used as a normal control. However, as demonstrated by Sorokin et al. (2023), such samples bear the imprint of the tumor’s systemic influence (the “field effect”) and thus cannot be considered fully adequate controls [2]. Furthermore, the authors showed that postmortem RNA degradation can, by itself, generate artifactual ‘differential’ expression profiles. This underscores the critical importance of accounting for the PMI factor and underscores the necessity of using, as a control, truly normal tissue from the same donor processed under identical postmortem conditions.

Tumor cells, unlike their normal histotypic counterparts, are indeed often characterized by constitutively high expression of various genes. This elevated basal transcriptional state, coupled with their characteristic adaptations to hypoxia and nutrient deficiency (such as the Warburg effect), may render them less sensitive to the additional stress imposed by the postmortem environment. Consequently, this could lead to distinct dynamics of the thanatotranscriptome in neoplastic tissue. To test this hypothesis, we investigated the expression dynamics of several key genes involved in homologous recombination, apoptosis regulation, and cell cycle control in paired samples of normal and tumor (chemically induced hepatocellular carcinoma) tissue from mouse livers over a 24-h postmortem interval.

The aim of the present study was to evaluate the temporal dynamics of expression of a panel of these genes in normal and tumorous mouse liver tissue after euthanasia and to identify genes most sensitive to postmortem stress. By conducting a controlled, paired comparison, this work seeks to address a significant methodological gap and provide essential data for refining biobanking protocols and interpreting transcriptomic data derived from postmortem oncological specimens.

## 2. Materials and Methods

### 2.1. Animal Model and Sample Collection

A chemically induced hepatocellular carcinoma (HCC) model was reproduced in male C57Bl/6 mice, obtained from the Federal Research Center, Institute of Cytology and Genetics of the Siberian Branch of the Russian Academy of Sciences, Novosibirsk, as previously described [18]. The study design was reviewed and approved by the Commission for the Control of the Maintenance and Use of Laboratory Animals of the Preclinical Research Center at the Central Research Laboratory, Siberian State Medical University (Protocol No. 1, 13 October 2023).

**Figure 1:**
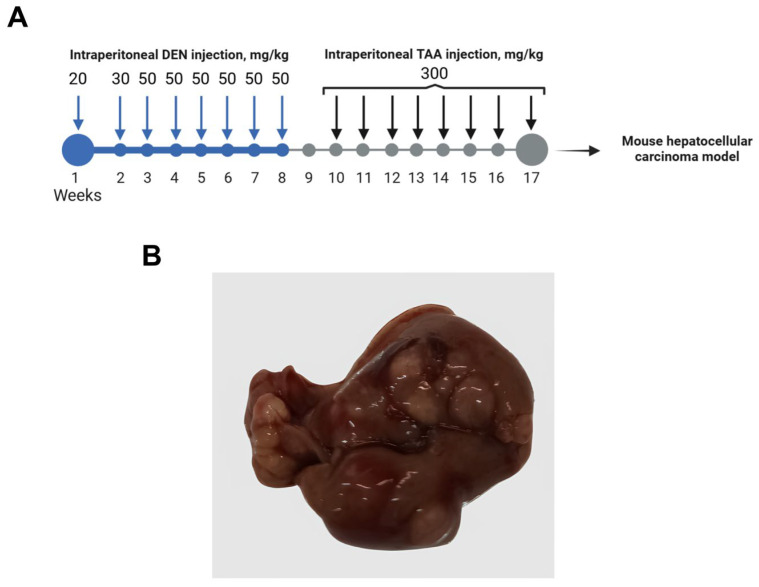
Experimental design and macroscopic pathology of the chemically induced hepatocellular carcinoma (HCC) model. (**A**) Schematic timeline of the carcinogenesis protocol; DEN—diethylnitrosamine, TAA—thioacetamide. (**B**) Representative macroscopic appearance of a liver from a carcinogen-treated mouse at the experimental endpoint.

Animals in the experimental group (*n* = 20) received sequential carcinogen administration. Starting from day 15 of life for a period of 8 weeks, diethylnitrosamine (DEN, Sigma-Aldrich, St. Louis, MO, USA) was administered weekly via intraperitoneal (i.p.) injection at increasing doses (20–50 mg/kg). This was followed by a 1-week washout period. Subsequently, for the next 8 weeks, thioacetamide (TAA, 300 mg/kg, Sigma-Aldrich, St. Louis, MO, USA) was administered i.p. twice a week. The solvent for both DEN and TAA was saline (0.9% NaCl). Control animals (*n* = 20) received saline injections according to the same schedule (Figure 1A).

Seven weeks after the final administration, animals were euthanized by cervical dislocation following anesthesia with an inhalational agent (Forane, AESICA QUEENBOROUGH Limited, Queenborough, Kent, UK). Immediately prior to euthanasia, animals were allocated into four groups (*n* = 5 per group) to simulate a gradually increasing postmortem interval (PMI), adapting to an established protocol [19]. The PMI simulation conditions were as follows: (1) immediate necropsy (0 h PMI); (2) storage of carcasses for 3 h at +22 °C (3 h PMI); (3) storage for 3 h at +22 °C followed by 9 h at +4 °C (12 h PMI); and (4) storage for 3 h at +22 °C followed by 21 h at +4 °C (24 h PMI).

Samples of normal liver tissue were collected from healthy control animals. Tumor tissue samples (~20–50 mm^3^) were excised from the largest, clearly distinguishable HCC nodules in the liver of carcinogen-treated mice. Tissue fragments were immediately placed in RNAlater solution (Ambion, Naugatuck, CT, USA) for subsequent RNA isolation and RNA integrity assessment (RNA Quality Number, RQN). For histopathological analysis, parallel tissue samples were fixed in 10% neutral-buffered formalin for 24 h.

### 2.2. RNA Isolation and Quality Assessment

Total RNA was isolated from the collected tissue samples using the RNeasy Plus mini Kit (Qiagen, Hilden, Germany) according to the manufacturer’s protocol. RNA concentration was measured using the Qubit 4.0 fluorometer (Thermo Fisher Scientific, Waltham, MA, USA) with the Qubit RNA HS Assay Kit (Thermo Fisher Scientific, Waltham, MA, USA). RNA concentrations ranged from 50 to 100 ng/µL. RNA integrity was assessed by capillary electrophoresis using the Tape Station 4150 system and the RNA ScreenTape analysis kit (Agilent Technologies, Santa Clara, CA, USA), yielding the RNA Quality Number (RQN) [20]. All isolated RNA samples were stored at −80 °C in a low-temperature freezer (Sanyo, Osaka, Japan) until further analysis.

### 2.3. Quantitative Real-Time PCR (RT-qPCR) Analysis

The expression levels of the target genes BRCA1, BRCA2, ATM, CDK12, CHEK1, and CHEK2 were evaluated using reverse-transcription quantitative real-time PCR (RT-qPCR) based on TaqMan chemistry on a Rotor-Gene-6000 thermocycler (Corbett Research, Rose Bay NSW 2029, Australia). Commercially synthesized primer and probe sets for the target genes (DNA-synthesis, Russia) were used to ensure high specificity and reproducibility.

The PCR reaction was performed in a final volume of 15 µL containing: 250 µM dNTPs (Sibenzyme, Novosibirsk, Russia), 300 nM of each forward and reverse primer, 200 nM of the TaqMan probe, 2.5 mMMgCl_2_, 1× SE buffer (67 mM Tris–HCl pH 8.8 at 25 °C, 16.6 mM (NH_4_)_2_SO_4_, 0.01% Tween-20), 2.5 units of HotStartTaq polymerase (Sibenzyme, Novosibirsk, Russia), and 50 ng of cDNA. The two-step thermal cycling protocol consisted of an initial denaturation at 94 °C for 10 min, followed by 40 cycles of denaturation at 94 °C for 10 s and combined annealing/extension at 60 °C for 20 s. All reactions were performed in triplicate.

Two reference genes, GAPDH and ACTB, were used for normalization. The relative expression of the target genes, expressed in arbitrary units, was calculated using the Pfaffl method [21]. RNA isolated from normal liver tissue collected immediately after euthanasia (0 h PMI) was used as the calibrator sample. This point served as the common baseline for the comparative expression analysis of all other samples, including both tumor tissue and normal tissue subjected to different storage intervals.

### 2.4. Histological Analysis

Tissue processing was performed according to standard histological protocols using an automated tissue processor, ASP 6025 (Leica Microsystems, Wetzlar, Germany), followed by paraffin embedding. Sections of 4–5 µm thickness were obtained from the paraffin blocks using an HM 430 sliding microtome (Thermo Fisher Scientific, Waltham, MA, USA). The sections were stained with hematoxylin and eosin using an automated Varistain™ Gemini slide stainer (Thermo Fisher Scientific, Waltham, MA, USA).

Morphological examination and photography of histological micropreparations were performed using a 3DHISTECH Pannoramic MIDI preparation scanner (3DHISTECH, Budapest, Hungary). The morphological assessment included evaluation of tissue changes for signs of autolysis.

### 2.5. Statistical Analysis

Data analysis was performed using Statistica 13.0 software (TIBCO Software Inc., Palo Alto, CA, USA). The normality of data distribution was assessed using the Shapiro–Wilk test. Since the distribution in the experimental groups deviated from normality, non-parametric statistical methods were applied. The Friedman test was used for comparisons across multiple related groups (time points), and the Mann–Whitney U test was used for pairwise comparisons between independent groups (normal vs. tumor tissue). Data are presented as the mean (M) ± standard deviation (SD). Differences were considered statistically significant at *p* < 0.05.

## 3. Results

### 3.1. Animal Hepatocellular Carcinoma Model

An autochthonous, chemically induced hepatocellular carcinoma (HCC) model was employed to avoid potential molecular artifacts associated with transplanted cell lines [18]. This syngeneic approach ensures that the observed transcriptomic dynamics are intrinsic to the tumor–host interaction and the postmortem environment, providing a clean background for comparative RT-qPCR analysis. The experimental design, including the carcinogen regimen, is summarized in Figure 1A.

Animals were monitored daily for overall health and signs of distress to ensure welfare throughout the carcinogenesis protocol. At the experimental endpoint, macroscopic examination of livers from carcinogen-treated animals revealed multiple tumor nodules, significant architectural distortion, and visible signs of fibrosis and steatosis (Figure 1B). These findings confirm successful induction of advanced neoplastic disease.

### 3.2. RNA Quality Dynamics During Simulated Postmortem Interval

One of the key objectives of this study was to assess the suitability of the biomaterial for molecular genetic analysis under simulated PMI conditions. Quality control of the isolated RNA showed that the 260/280 absorbance ratio consistently remained within the optimal range of 2.0–2.3 at all experimental stages. However, with increasing PMI, the RQN value showed a sequential decline in both normal and tumor tissue samples (Table 1).

**Figure 2:**
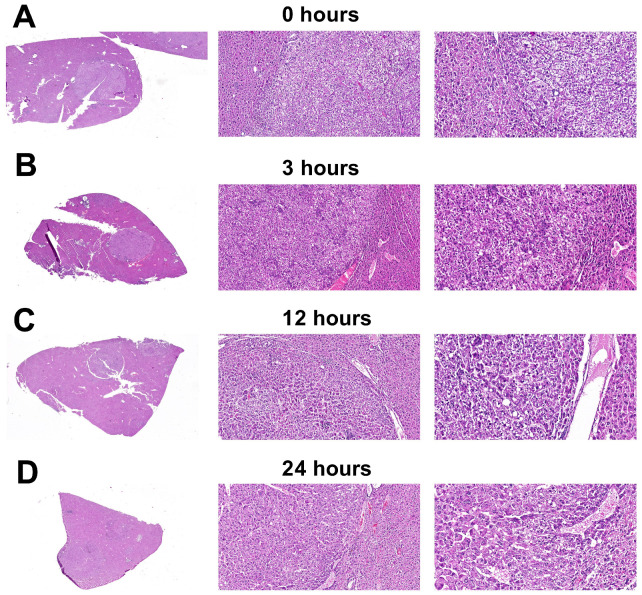
Histological appearance of liver tissue from mice with chemically induced hepatocellular carcinoma, sampled at different postmortem intervals: 0 h (**A**), 3 h (**B**), 12 h (**C**) and 24 h (**D**). Hematoxylin and eosin staining. Magnification in each image set from left to right: ×1, ×10, and ×20.

In parallel, histopathological examination with hematoxylin and eosin staining was performed to confirm tumor presence and to assess gross morphological changes; no progressive autolysis was observed at the light microscopy level across the studied PMI (Figure 2).

In the liver tissue of control animals, the RQN decreased by 27% at the maximum PMI duration (24 h post-euthanasia) (*p* = 0.0020). In tumor samples, RNA degradation was slightly more pronounced, with an RQN drop of 32% (*p* = 0.0003). Despite this reduction, since an RQN threshold of ≥5.0 is accepted as a criterion for sample suitability in biomedical research [22], all data obtained in subsequent experiments can be considered adequate across all PMI time points.

### 3.3. Histopathological Characterization and Stability of Tissue Morphology

Morphological analysis of liver samples from experimental animals collected at all time points revealed a consistent and reproducible pattern of tumor growth (Figure 2). In all examined specimens, the tumor tissue presented with a well-defined border against the non-neoplastic liver parenchyma. The predominant histological architecture of the neoplasm was a trabecular pattern, formed by polymorphic tumor cells exhibiting marked nuclear atypia. Characteristic cytological features included clumped chromatin and the presence of distinct, small nucleoli in a subset of cells. A pathognomonic finding was the presence of cytoplasmic hyaline eosinophilic inclusions, detectable in the majority of tumor cells. The tumor stroma was scant and contained few, dilated blood vessels. This morphological picture allowed for the verification of the process as hepatocellular carcinoma.

Notably, as illustrated in Figure 2 (panels A–D for 0, 3, 12, and 24 h, respectively), the tissue morphology remained stable across the PMI, without discernible signs of progressing autolysis. This morphological stability stands in contrast to the dynamic molecular processes recorded at the level of gene expression.3.4. Postmortem Transcriptional Dynamics in Normal and Tumor Tissues

**Figure 3:**
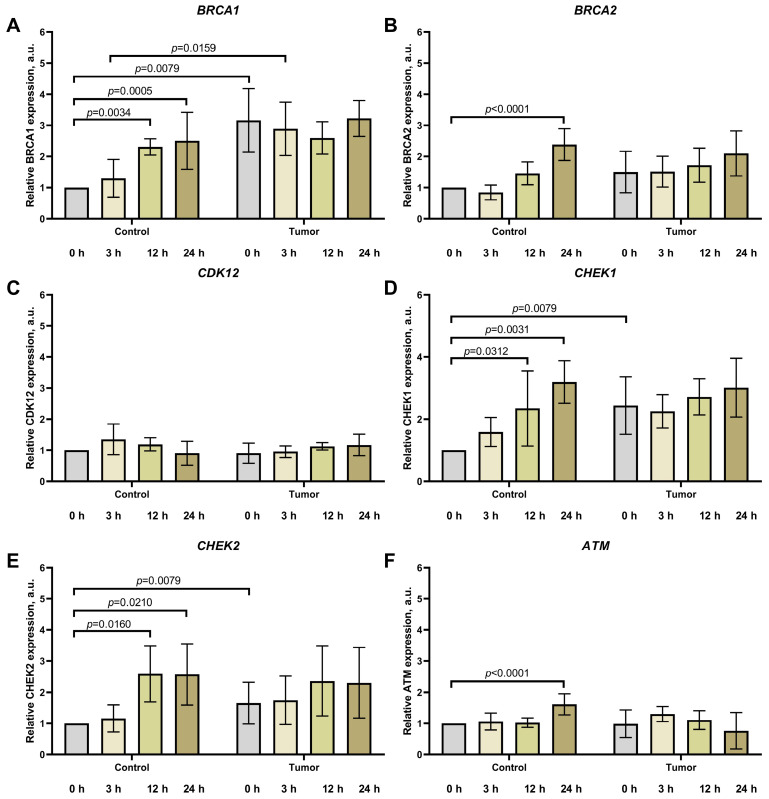
Expression levels of homologous recombination genes BRCA1 (**A**) and BRCA2 (**B**), cell cycle regulators CDK12 (**C**), CHEK1 (**D**), CHEK2 (**E**), and the apoptosis-related gene ATM (**F**) in normal and tumor mouse liver tissue at various times after euthanasia. For the first three hours post-euthanasia, carcasses were kept at room temperature, then transferred to a refrigerator (+4 °C). Each group included *n* = 5 animals. Data are presented as mean ± SD (M ± SD).

**Figure 4:**
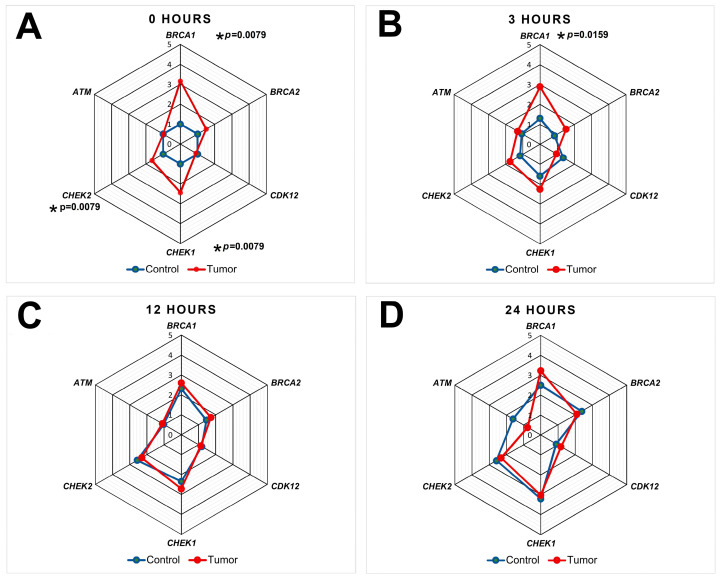
Changes in the expression of BRCA1, BRCA2, CDK12, CHEK1, CHEK2, and ATM genes in normal and tumor mouse liver tissue with increasing postmortem interval: (**A**) 0 h, (**B**) 3 h, (**C**) 12 h and (**D**) 24 h. For the first three hours post-euthanasia, carcasses were kept at room temperature, then transferred to a refrigerator (+4 °C). Each group included *n* = 5 animals. An asterisk (*) indicates a statistically significant difference with marked *p*-value in gene expression between tumor and control (normal) tissue at a given time point.

Analysis of gene expression related to homologous recombination, cell cycle regulation, and apoptosis revealed substantial differences in the dynamics between normal and tumor liver tissues depending on the postmortem interval (PMI) (Figure 3 and Figure 4).

In normal liver tissue, a pronounced increase in the expression of most studied genes was observed. The mRNA level of BRCA1 rose more than 2.5-fold by 24 h PMI (to 2.51 ± 0.82 a.u., *p* = 0.0005), with a significant increase already evident at 12 h of postmortem storage (2.31 ± 0.23 a.u., *p* = 0.0034). BRCA2 showed the highest fold change, increasing 2.4-fold by 24 h to 2.39 ± 0.42 a.u. (*p* < 0.0001). CHEK1 and CHEK2, which play key roles in cell cycle checkpoint control, exhibited expression increases of 3.2-fold and 2.6-fold, respectively, by the end of the experimental period (3.19 ± 0.56 a.u., *p* = 0.0031 and 2.60 ± 0.88 a.u., *p* = 0.0210).

While saline injection (carcinogen vehicle) may induce minimal cellular stress, any effects on gene expression are generally considered negligible and not biologically significant. Furthermore, the control group in our study served to monitor spontaneous tumor formation or vehicle-induced changes, and none were observed. Importantly, to the best of our knowledge, there is no scientific evidence indicating that saline per se has any meaningful impact on the expression of the genes investigated here.

In tumor tissue, a fundamentally different pattern characterized by transcriptional stability was observed with increasing PMI. To statistically confirm the transcriptional stability in tumor tissue, we compared expression levels at 3, 12, and 24 h PMI against the 0 h time point. The initially elevated expression of BRCA1 (3.16 ± 0.91 a.u.), CHEK1 (2.44 ± 0.83 a.u.), and CHEK2 (1.65 ± 0.60 a.u.) in the livers of mice with hepatocellular carcinoma remained at a high level throughout the entire observation period without significant changes. The transcriptional activity of BRCA2, CDK12, and ATM also remained virtually unaltered across all selected time points.

Comparative analysis revealed a critical pattern: the initially pronounced differences in the expression of individual genes between normal and tumor tissue were negated at later PMI time points. For instance, while BRCA1 expression in the tumor was 3.2-fold higher than in normal tissue immediately after euthanasia (*p* = 0.0079), this difference was no longer detectable by 24 h. A similar convergence of expression levels between normal and tumor tissues was observed for the genes BRCA2, CHEK1, and CHEK2 by 12–24 h PMI.

Thus, under conditions of a prolonged postmortem interval, a leveling of expression levels for DNA repair and cell cycle regulation genes occurs between normal and tumor liver tissues.

## 4. Discussion

The results obtained in the present study demonstrate fundamental differences in the transcriptional response of normal and tumor liver tissue to postmortem stress, which has significant implications for interpreting data derived from autopsy and biobanked material. A key observation is the phenomenon of transcriptional stability for genes involved in DNA homologous recombination and cell cycle regulation in chemically induced hepatocellular carcinoma tissue, contrasting with the significant dynamics of their expression observed in normal liver parenchyma over a 24-h PMI.

Our data are consistent with the actively developing paradigm of thanatotranscriptomics, which posits that organismal death initiates a cascade of coordinated changes in gene expression [6,7,8]. The observed 2.4- to 3.2-fold increase in transcript levels of key homologous recombination (BRCA1, BRCA2) and cell cycle regulation (CHEK1, CHEK2) genes in normal liver tissue over the 24 h following euthanasia can be viewed as an adaptive cellular response to the critical damage induced by ischemia and hypoxia. A similar postmortem activation of survival and repair mechanisms has been previously described in human blood [6] and skeletal muscle [14], where the induction of genes associated with the response to oxidative stress and hypoxia was noted. It is plausible that normal tissue attempts to counteract the postmortem stress of the organism by activating conserved protective pathways in this manner. However, this conclusion is not unequivocal, as simultaneous activation of pro-apoptotic and suppression of anti-apoptotic genes has been demonstrated in postmortem human liver tissues [15].

In contrast, the tumor tissue in our study demonstrated remarkable transcriptional resilience. Genes BRCA1, BRCA2, CHEK1, CHEK2, and ATM did not show substantial changes in expression throughout the increasing PMI, while the initially elevated basal mRNA levels of BRCA1, CHEK1, and CHEK2 in the tumor remained stably high. This aligns with fundamental features of cancer biology, which include the constitutive activation of multiple survival signaling pathways, impaired apoptosis, and chronic genomic stress [2,17]. Consequently, the transcriptomic landscape of tumor tissue exists in a hyperactivated state, rendering it less variable compared to normal tissue.

Recent evidence from other tissue types suggests that reference transcripts can remain stable for extended periods under controlled conditions [23]. Future studies incorporating longer PMIs will be necessary to establish the full window of suitability for tumor tissue in biobanking applications.

On the other hand, the observed increase in expression in normal tissue may, with high probability, result from the differential degradation of messenger RNAs. This hypothesis is supported by studies showing that postmortem RNA degradation is selective, exhibiting tissue- and gene-specific patterns heavily dependent on transcript length, secondary structure, and the presence of specific motifs recognized by RNases [3,4,24]. From this perspective, the contrast between the rising expression of certain genes in normal tissue and the maintained stability in tumor samples could be explained by differences in the decay gradients of mRNAs. In tumor tissue, with its dysregulated metabolism and constitutively high stress levels [2,25], the studied genes likely share a similarly enhanced resistance to degradation, or their degradation occurs at comparable rates, manifesting as transcriptional stability. Furthermore, the observed stability in the expression levels of the studied genes may also be attributed to the fact that tumor cells, due to metabolic reprogramming (the Warburg effect), possess greater inherent resistance to hypoxia during life than normal cells [26]. Indeed, a recent study by Choi et al. [23] demonstrated that the stability of individual transcripts varies significantly between genes and tissues, with some housekeeping genes (e.g., Gapdh, 5S rRNA) exhibiting remarkable stability over extended PMIs, while others (e.g., Sort1) degrade rapidly. These findings highlight the need for careful selection of reference genes and underscore that transcript stability is an inherent property influenced by both sequence and cellular context. Furthermore, ischemic tolerance varies considerably across organs, as illustrated by the differential morphological and molecular changes observed in brain versus skeletal muscle [23]. A comparative analysis across organs with varying sensitivities to ischemia would therefore provide valuable insights for optimizing tissue handling protocols in biobanking.

A key conclusion drawn from the analysis of the presented experimental results is the convergence of transcriptional profiles between normal and tumor tissue at later PMI time points. The initially substantial differences in the expression of BRCA1, CHEK1, and CHEK2 between the groups were completely eliminated by 12–24 h PMI. This creates a serious risk of erroneous data interpretation when using samples with a prolonged PMI, as fundamental in vivo differences can be entirely masked. Importantly, these processes occur against a backdrop of formally adequate biomaterial quality [22,27], while routine histological analysis did not reveal increasing autolysis. To elucidate the patterns of ultrastructural changes, studies employing electron microscopy techniques are warranted [28]. Consequently, standard quality control protocols for biobanking procedures and deferred molecular–biological investigations may be insufficient for detecting profound distortions in expression patterns caused by thanatotranscriptomic shifts or differential transcript degradation. Our data are consistent with the findings of Sorokin et al. [2], where postmortem RNA degradation was shown to generate differential expression profiles, further underscoring the critical importance of accounting for PMI.

Our results demonstrate that the resilience of the transcriptomic profile to postmortem stress is an intrinsic and significant tissue characteristic. The identified patterns of postmortem gene expression in normal and tumor mouse tissues highlight the necessity for strict tracking and minimization of PMI during the collection of biological samples for molecular studies, as well as the importance of using genuine normal tissue as a control [2,8]. A major strength of this work is the direct, controlled comparison of gene expression between normal liver tissue (from vehicle-treated control animals) and chemically induced HCC tissue, allowing for the assessment of tumor-specific postmortem changes without interference from xenograft or cell-line artifacts. The integrated analysis of gene activity in collected samples and the stability of their transcripts under postmortem stress requires further investigation and opens up a new, promising direction within thanatotranscriptomics and the biobanking of oncological specimens.

## 5. Conclusions

This study, investigating the dynamics of gene expression profiles, revealed fundamental differences in the transcriptional response of normal and tumorous liver tissue from mice with experimental hepatocellular carcinoma to postmortem stress. In tumor tissue, the expression of DNA repair (BRCA1, BRCA2), cell cycle control (CHEK1, CHEK2), and apoptosis-related (ATM) genes did not undergo significant changes over a 24-h PMI, whereas their expression increased markedly in normal liver tissue.

The observed convergence of transcriptional profiles, whereby initially substantial differences in the expression of individual genes between control and tumor groups were eliminated by later PMI time points, is a critical finding. This phenomenon develops despite formally adequate RNA quality and the absence of clear signs of progressive autolysis in histology, establishing the resilience of the tumor tissue transcriptomic profile to postmortem stress as an independent and important tissue characteristic.

The obtained data suggest that standard quality criteria, such as RNA integrity (RQN) and histological analysis, may be insufficient for detecting significant distortions in expression patterns induced by postmortem changes.

Thus, the resilience of the transcriptome to postmortem stress differs fundamentally between normal and neoplastic liver parenchyma. These findings have direct implications for biobanking practices and for the interpretation of transcriptomic data derived from autopsy specimens. The use of normal tissue as a comparator for tumor samples requires strict control of the postmortem interval, as otherwise biologically significant differences may be masked by postmortem transcriptional changes. The differential stability of the transcriptome between normal and tumor tissue underscores the need for careful selection of control tissues in oncological research, particularly when working with biobanked material collected under varying PMI conditions.

## Figures and Tables

**Table 1:** RNA Integrity (RNA Quality Number, RQN) in Normal and Tumor Mouse Liver Tissues at Different Postmortem Intervals (PMI). Data are presented as mean ± SD (M ± SD).

PMI, h	RNA Quality Number	
	Control	Tumor
0	8.97 ± 0.65 *p*_0–3_ = 0.4523 *p*_0–12_ = 0.0502 *p*_0–24_ = 0.0020	8.70 ± 0.61 *p*_Control-Tumor_ = 0.8413 *p*_0–3_ = 0.5228 *p*_0–12_ = 0.0013 *p*_0–24_ = 0.0003
3	8.23 ± 0.89 *p*_3–12_ = 0.2534 *p*_3–24_ = 0.0277	8.07 ± 0.86 *p*_Control-Tumor_ = 0.8413 *p*_3–12_ = 0.0213 *p*_3–24_ = 0.0005
12	7.49 ± 0.82 *p*_12–24_ = 0.1634	6.32 ± 0.87 *p*_Control-Tumor_ = 0.0556 *p*_12–24_ = 0.4862
24	6.57 ± 0.85	5.92 ± 0.70 *p*_Control-Tumor_ = 0.4206

## Data Availability

The data presented in this study are available on reasonable request from the corresponding author (E.V.U.) due to legal restrictions.
